# The benefits and acceptability of virtual reality interventions for women with metastatic breast cancer in their homes; a pilot randomised trial

**DOI:** 10.1186/s12885-021-09081-z

**Published:** 2022-04-02

**Authors:** Lisa M. Reynolds, Alana Cavadino, Stanley Chin, Zoë Little, Amelia Akroyd, Geraldine Tennant, Rosie Dobson, Reuben Broom, Adèle Gautier

**Affiliations:** 1 Department of Psychological Medicine, The University of Auckland, Auckland, New Zealand; 2 Section of Epidemiology and Biostatistics, The University of Auckland, Auckland, New Zealand; 3 School of Psychology, Victoria University of Wellington, Wellington, New Zealand; 4 National Institute for Health Innovation, The University of Auckland, Auckland, New Zealand; 5 Medical Oncology Department, Auckland City Hospital, Auckland, New Zealand; 6 Breast Cancer Foundation NZ, Auckland, New Zealand

**Keywords:** Advanced cancer, Intervention, Pain, Fatigue, Quality of life

## Abstract

**Background:**

Women with metastatic breast cancer (MBC) report debilitating physical and psychological symptoms, including fatigue, anxiety, and pain, that greatly impact their quality of life. Immersive virtual reality (VR) has been proposed as an adjunctive pain therapy for patients with cancer, and evidence suggests it may also decrease symptoms of anxiety and depression. The purpose of this pilot study was to assess whether VR should be pursued as a feasible and acceptable adjunctive therapy to alleviate physical and psychological symptoms in women with MBC.

**Methods:**

We conducted a pilot study testing the acceptability and efficacy of VR interventions with MBC patients to improve quality of life and to produce enduring decreases in fatigue, pain, depression, anxiety, and stress. Participants completed two different week-long VR experiences, reporting the prevalence of symptoms immediately before and after each study week, and 48 h later. Linear mixed models including fixed effects (VR intervention, counterbalancing order, and study week) and random effects (participant) were used to assess the effect of immersive VR on all outcome measures.

**Results:**

Thirty-eight women with MBC completed the VR interventions and were included in analyses. Significant improvements post-intervention and/or 48 h later were demonstrated for quality of life, fatigue, pain, depression, anxiety, and stress. Across the entire study period, these differences met the criteria of a clinically important difference for quality of life, fatigue, depression, and stress. Participants reported feelings of relaxation and enjoyment and were highly likely to use the interventions gain.

**Conclusions:**

Our results demonstrate that VR experiences offer enduring benefits to the physical and psychological well-being of women with MBC. VR interventions are a feasible and acceptable intervention that can be conducted in a patient’s own home. Such interventions are worthy of future investigation as a novel approach to improving quality of life in a patient population that have often been overlooked.

**Trial registration:**

Prospectively registered on 25th October 2019 with Australian New Zealand Clinical Trials Registry (ref: ACTRN12619001480178).

## Background

Metastatic Breast Cancer (MBC) can present de novo (5–10% of all breast cancer diagnoses), or as a recurrence after early breast cancer (20–30% of patients will experience a recurrence) [1]. MBC is essentially incurable and treatment for patients focuses on prolonging survival and preserving quality of life [2]. People living with MBC commonly report experiencing debilitating symptoms from both the cancer itself and its treatments [3]. Symptoms are both physical (e.g., pain, fatigue [4]) and psychological (e.g., depression, anxiety [5]) and can significantly impact quality of life and be a source of suffering and disability [4]. Common treatments to address such symptoms include rehabilitation programmes [6] and psychosocial interventions [7], however treatments can be resource intensive, such that novel, pragmatic, approaches are required.

Immersive virtual reality (VR) has been proposed as an adjunctive therapy for reducing cancer-related symptoms [8]. VR presents an interactive 3D human-computer interface that allows individuals to interact with and become immersed in a computer-generated environment in a naturalistic fashion [9]. Immersive VR uses a head-mounted display which blocks the participants’ view of the real world and often excludes sounds via noise-cancelling headphones, creating the illusion of being present in a virtual world [8, 9]. An example of an immersive VR application is ‘Snow World’ in which patients take a virtual journey down an icy river [10]. This application has been shown to effectively reduce pain in patients undergoing burn wound debridement [10]. VR has also been shown to decrease other physical symptoms such as vomiting and nausea [11], and even alter the perception of the length of one’s treatment [12]. In addition to physical symptoms, VR interventions effectively reduce psychological symptoms such as anxiety [11] and increase positive mood states and induce states of relaxation [13]. These beneficial effects have been demonstrated in a variety of clinical applications including during chemotherapy [11], painful procedures [14], and hospitalisation [13]. The effects do not appear to diminish with repeated use [15], meaning it can be used repeatedly throughout treatment. Thus, VR has been shown to effectively reduce certain symptoms in clinical contexts.

However, the literature so far is limited in several ways. First, studies have tended to focus on the ability of VR to reduce a single symptom such as pain or anxiety and ignored more holistic aspects of health status such as quality of life. Patients with MBC have marked impairments to quality of life [16] and it is important to consider such aspects of well-being in the assessment of interventions. Second, while pain has received considerable attention, other symptoms shown to impact this population have been relatively ignored. For example, fatigue is difficult to treat [17] and is rated by MBC patients as the symptom with the most impact on quality of life [18]. Apart from a single early study which found that the “Bedside Wellness System” VR application for cancer patients significantly decreased fatigue [19], no other studies have investigated the effect of VR on fatigue. Third, no studies of immersive VR for cancer patients have been conducted outside of a medical or laboratory environment. The focus on treatment-associated problems and the financial costs associated with VR mean that previous studies have been primarily hospital-based. However, the recent introduction of more affordable VR devices allows for home-based research where the patient may operate the intervention themselves [13]. The portability of such an intervention is also potentially of benefit to patients who face barriers accessing standard support services.

Finally, and arguably most importantly in the case of chronic disease, it has not been adequately established whether the benefits of VR can endure for longer timespans beyond usage of the device. There are anecdotal reports from chronic pain patients that analgesia following VR exposure can last for hours or days after the session ends [20], however, work with cancer patients have primarily focused on in-the-moment distraction of treatment [12] rather than determining whether positive benefits might be maintained. More systematic research is needed to establish whether anecdotal reports are accurate and translate to cancer contexts.

Here, we report a pilot study testing the efficacy and acceptability of a home-based immersive VR intervention to produce sustained improvements in quality of life and reductions in fatigue, pain, depression, and anxiety in patients with MBC. The primary aim of this pilot study was to establish whether VR should be pursued as a feasible and acceptable adjunctive therapy for producing enduring, clinically meaningful, alleviation of physical and psychological symptoms experienced by women with MBC.

## Methods

### Design

A mixed-methods, crossover design integrated pre-intervention, post-intervention, and 48-h follow-up assessments of scores on outcome measures. Participants completed two different VR interventions, the order of which was counterbalanced and randomised between groups. The study was prospectively registered on the Australian New Zealand Clinical Trials Registry (ref: ACTRN12619001480178) and ethical approval was granted by the New Zealand Health and Disability Ethics Committee (ref: 19/NTB/146). All participants gave informed consent to participate.

### Participants

Women with MBC were invited to participate through the mailing lists of Breast Cancer Foundation NZ (BCFNZ) and Sweet Louise (a charity support service for people with MBC). Given the exploratory nature of this study, a sample of 30 participants was determined as sufficient to assess the acceptability of a trial of this nature and to identify a large effect in outcomes. Participants were required to have a diagnosis of MBC, be over 18 years, be able to physically wear and tolerate the VR headset, and have experienced symptoms of fatigue, pain, or anxiety in the week prior to enrolment by study researchers. Participants were excluded if they had any visual, hearing, or cognitive impairments that would limit their ability to take part in the study, or if they could not read, speak, or write in English. Participants received NZ$100 for their participation.

### VR intervention

**Fig. 1:**
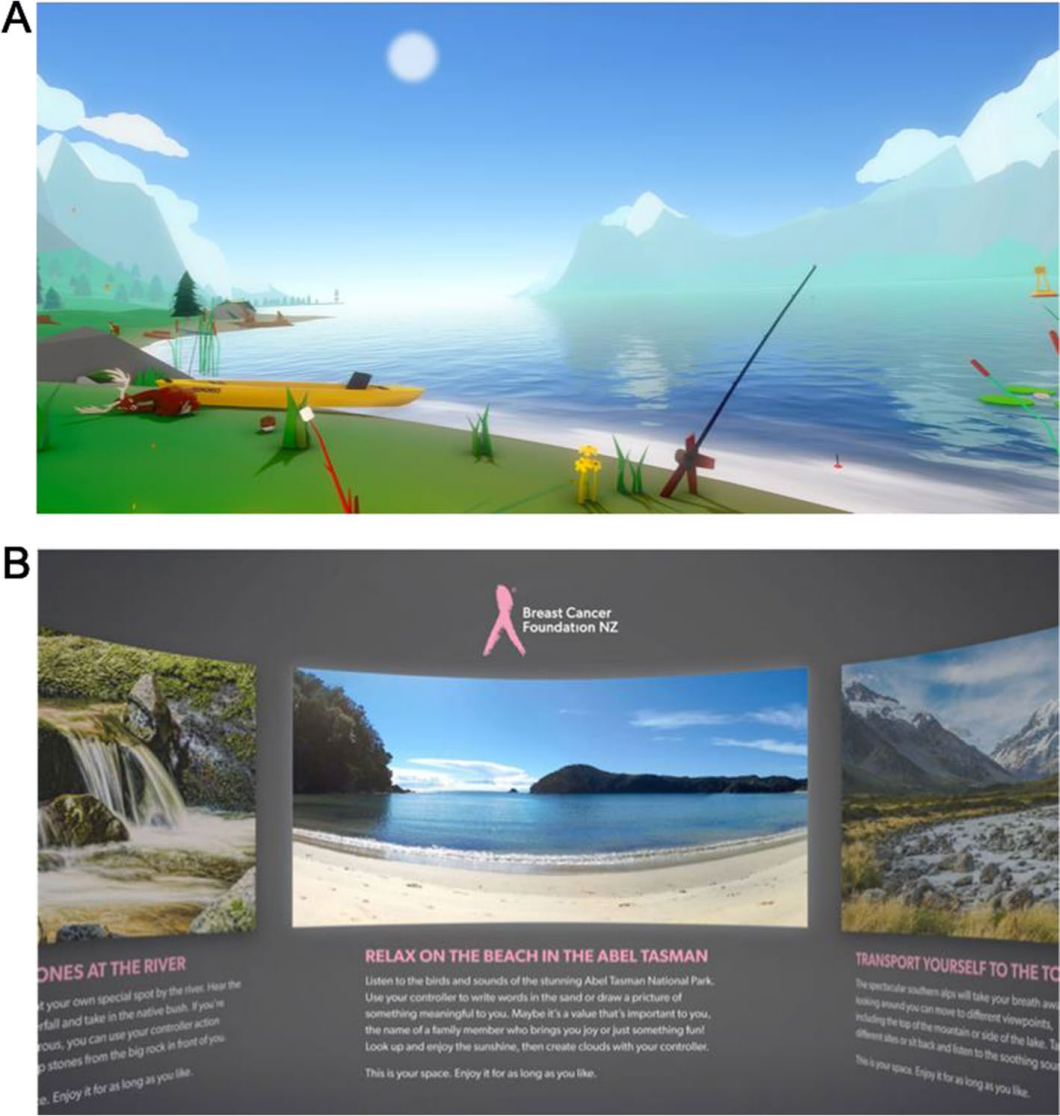
Example Screen Captures from the Happy Place and Ripple VR Experiences. (**A**) a scene from the Happy Place VR experience. (**B**) the menu in the Ripple VR experience, where participants can choose between the river (left) beach (middle) or mountain (right) experiences

All participants were provided with a Pico Goblin VR headset for use in their own homes. This included a 1280 × 1440 px LCD screen with a refresh rate of 70 Hz and a controller. Participants also received headphones (Panasonic RP-HT161), written instructions, and copies of the questionnaires. Two different VR interventions, “Happy Place” and “Ripple”, were used. Screen captures from each experience are provided in Fig. 1.

Happy Place [21], developed by Hjärtat in 2016, is a commercially available VR application in which participants experience a tranquil, animated camping scene. Participants experience changes to the weather and time of day and can interact with optional tasks, guided relaxation, and soothing music. Ripple is a collection of three short 360° VR nature scenes commissioned by BCFNZ and developed by Mixt Studio [22]. The three experiences are as follows: 1) a beach where the participant can write words in the sand or the sky; 2) a waterfall where the participant can stack stones; and 3) a mountain range where the participant can jump between different locations amongst mountaintops and lakes. The usability and acceptability of the Ripple VR experience in women with MBC had been found to be high in a previous proof-of-concept study.

### Outcome measures

**Fig. 2:**
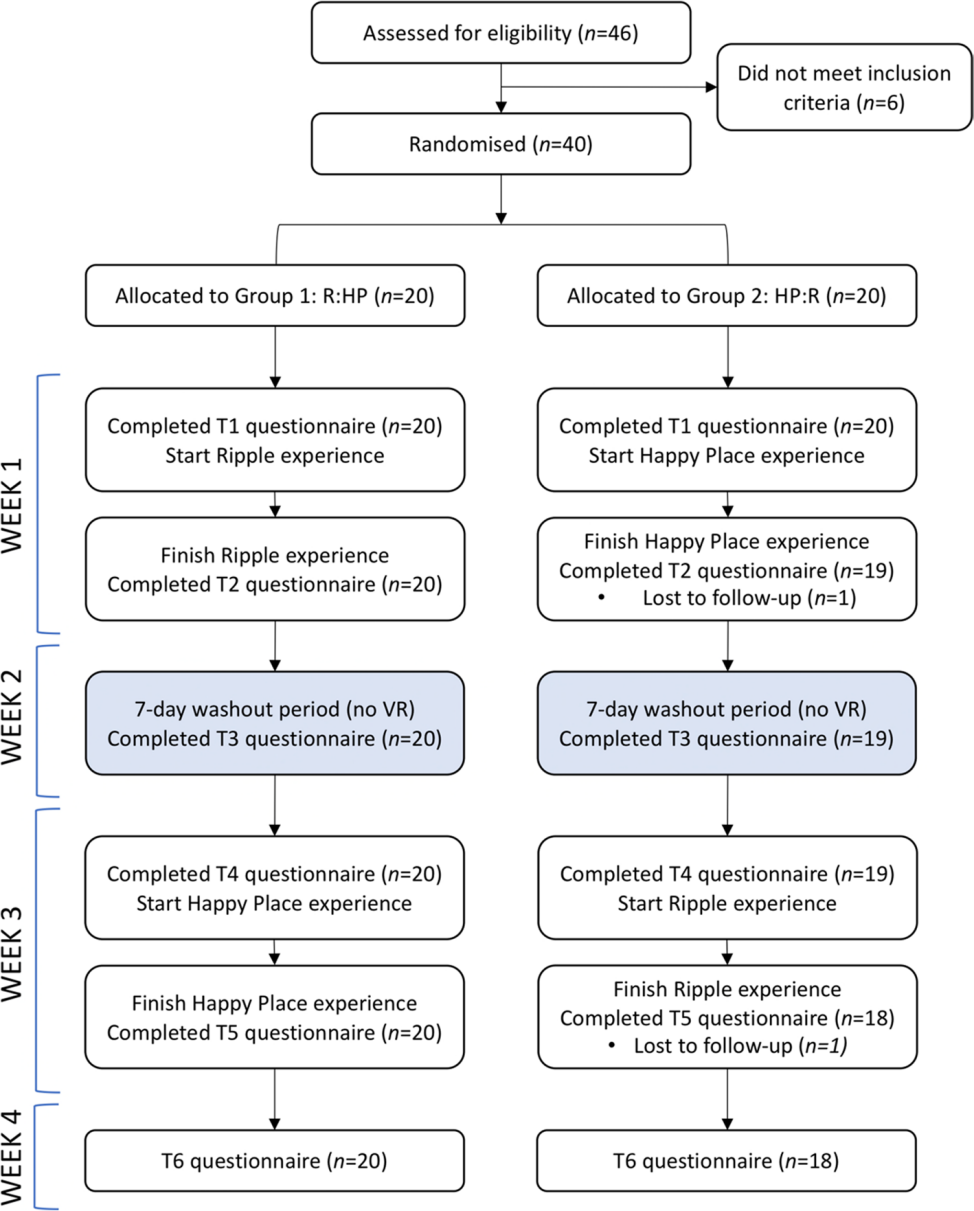
Procedural Diagram of Enrolment, Randomisation, Allocation, and Intervention

Participants completed questionnaires online at six different time points (See Fig. 2), each taking approximately 15–20 min to complete. Table 1 provides a summary of measures, the timing of application, and the minimum clinically important difference (MCID) as reported in the relevant literature.

**Table 1:** Summary of Outcome Measures

Measure	Direction of Score	# of Items	Timepoints Administered	Minimum Clinically Important Difference	Possible Range
**Primary Outcome Measure**					
EQ-5D-5L Index	↑ = better	5	T1, T2, T4, T5	+ 0.08 [23]	0–1
EQ-FD-5 L VAS	↑ = better	5	T1, T2, T4, T5	+ 7 [23]	0–100
**Secondary Questionnaires**					
FACIT Fatigue	↑ = worse	13	T1, T2, T3, T4, T5, T6	−4 points [24]	0–52
BPI	↑ = worse	11	T1, T2, T3, T4, T5, T6	Cohen’s *d* = 0.50 [25]	0–110
DASS-21 Total	↑ = worse	21	T1, T2, T3, T4, T5, T6		0–63
DASS Depression	↑ = worse	7	T1, T2, T3, T4, T5, T6	−5.01 points [26]	0–21
DASS Anxiety	↑ = worse	7	T1, T2, T3, T4, T5, T6	−5.38 points [26]	0–21
DASS Stress	↑ = worse	7	T1, T2, T3, T4, T5, T6	−4.90 points [26]	0–21

Our primary outcome measure was quality of life as measured by the EQ-5D-5L [27]. The EQ-5D-5L asks participants to indicate their functioning in five areas – mobility, self-care, usual activities, pain/discomfort, and anxiety/depression. Participants rate themselves as having no problems, slight problems, moderate problems, severe problems, or extreme problems. The EQ-5D-5L has demonstrated good construct validity and reliability with cancer patients [23]. We used an online calculator (from euroqol.org) and UK values to calculate each participant’s index score. Participants also rate their quality of life on a visual analogue scale (VAS). In the current study, the index had an average Cronbach’s alpha across timepoints of 0.77 (*SD* = 0.08).

The Functional Assessment of Chronic Illness Therapy Fatigue scale (FACIT-Fatigue) [28] measures cancer-related fatigue. Participants rate how true items such as “I feel fatigued” have been over the last 7 days on a 5-point Likert scale from 0 (*not at all*) to 4 (*very much*) and scores are summed across the 13 items. Previous work has demonstrated the FACIT-Fatigue has good test-retest reliability, internal consistency, and concurrent validity in cancer patients [29]. The scale had good reliability in the current work across all timepoints (average Cronbach’s alpha = 0.90, *SD* = 0.03).

The Brief Pain Inventory – Short Form (BPI) includes 11 items used to assess pain. Participants rate the severity of their pain over the last 24 h and how it has interfered with things such as “enjoyment of life” on a scale from 0 (*no pain/does not interfere*) to 10 (*pain as bad as you can imagine/interferes completely*). Item scores are summed to create a total. The BPI has been shown to have excellent internal consistency among cancer patients [30] and was similarly observed in the current work with Cronbach’s alpha across timepoints of 0.94 (*SD* = 0.01).

The 21-item short version of the Depression, Anxiety, and Stress Scales (DASS-SF) [31] was used to measure depression, anxiety, and stress in our sample. The DASS-21 has been widely used in cancer patients and been shown to be valid and reliable [32]. This measure includes a subscale of 7 items for each construct. Participants rate the severity of negative emotions they experienced in the previous week on a scale of 0 (*did not apply to me at all*) to 3 (*applied to me very much, or most of the time*). Items are summed to create three separate scores for depression, anxiety, and stress. The depression subscale had an average reliability of 0.85 (*SD* = 0.07), anxiety was 0.51 (*SD* = 0.14), and the stress subscale average was 0.82 (*SD* = 0.05). Due to the low internal reliability of the anxiety scale, we removed one item (item 2, “mouth dryness”) as this is also a common side effect of chemotherapy and analgesic medicines prescribed in MBC. After removing this item, the average Cronbach’s alpha of the anxiety subscale improved to 0.65 (*SD* = 0.10).

Along with these questionnaires, the baseline (T1) questionnaire asked demographic and clinical questions. Finally, at the end of each intervention (T2 and T5), acceptability and satisfaction with the intervention were assessed by asking participants how likely they would be to use the intervention again (rated 0 to 100), and open-ended questions about what they liked and did not like about the interventions and suggestions for improvement.

### Procedure

Participants were block randomised by age (< 50 years vs. 50 years+) to order of the VR interventions. An independent researcher created and uploaded a randomisation table to REDCap. The study researchers were blinded to randomisation until after REDCap had allocated participants to group. Group 1 used Ripple before Happy Place (R:HP), and Group 2 experienced Happy Place before Ripple (HP:R). There was a one-week washout period to minimise carryover effects between the two interventions (see Fig. 2). During the intervention weeks, participants were instructed to use the VR experience every day for a minimum of 10 min. Participants kept a written log of the time spent using the headset to check adherence; these records were used to summarise participants’ engagement with VR.

### Statistical analyses

We tested the normality of all continuous questionnaire data at each time point visually and with Shapiro-Wilk tests. The normality of quality of life measures (EQ-5D-5L index and VAS) were improved when squared, and the normality of the DASS measures (total, depression, anxiety, and stress) were improved with square-root transformation. The transformed data was therefore used for all analyses for these measures. To examine the effect of the VR interventions on our various outcome measures, linear mixed models were used, including fixed effects of VR experience (as per assigned group of Ripple or Happy Place), time point (pre, post, or follow-up), study period (first or second intervention received), and interaction terms of time point with each period and VR experience. The REML method was used with a random effect term included for participant and with an autoregressive correlation structure used for residuals. REML estimation was used for all models, since maximum likelihood estimation can produce biased estimates of covariance parameters [33, 34]. The Kenward and Roger adjustment was also used, which can control type I errors in smaller samples [35]. Results are reported using pairwise comparisons of estimated marginal mean differences between study time points (pre vs post, post vs follow-up, pre vs follow-up). Residuals from all models were evaluated for normality and constant variance. Statistical significance was defined using a *p*-value < 0.05. Analyses were performed using SPSS (version 26) or Stata (version 16). We first looked at the average decrease across interventions by using the difference between the raw pre-intervention scores (average of T1 and T4) and follow-up scores (average of T3 and T6). For quality of life, we compared pre-intervention scores to the post-intervention score (the average of T2 and T5) as this was not measured at follow-up. The raw (untransformed) mean differences were calculated to enable comparisons with MCIDs and assessed according to the conventions summarised in Table 1. Thematic analysis was used to analyse open-ended questions regarding intervention acceptability.

## Results

Recruitment commenced in October 2019 and data collected until March 2020. Response to invitations exceeded expectations; 46 participants were initially assessed of whom 40 met the inclusion criteria (See Fig. 2 for the study flow diagram). Two participants were lost due to attrition over the course of the study, leaving a final sample size of 38. The remaining sample were primarily NZ European (81.6%, *n* = 31) with low representation from other ethnicities (see Table 2). The sample was well-educated with many who had completed tertiary education (57.9%, *n* = 22).

**Table 2:** Demographic Characteristics of the Sample

		Mean (SD) or N (%)		
Measure		All Participants (*N* = 38)	R:HP (*n* = 20)	HP:R (*n* = 18)
Age	Years; mean (SD)	52.03 (11.40)	52.70 (13.20)	51.28 (9.32)
Ethnicity	NZ European	31 (81.6%)	15 (75%)	16 (89%)
	NZ Maori	1 (2.6%)	0 (0%)	1 (5.6%)
	NZ Maori/European	5 (13.2%)	5 (25%)	0 (0%)
	Pacific	1 (2.6%)	0 (0%)	1 (5.6%)
Highest education	Secondary	16 (42.1%)	12 (60%)	4 (21%)
	Tertiary	15 (39.5%)	6 (30%)	9 (52.6%)
	Post-graduate	7 (18.4%)	2 (10%)	5 (26.3%)
Employment status	Full-time	10 (26.3%)	4 (20%)	6 (36%)
	Part-time	7 (18.4%)	5 (25%)	2 (11%)
	Not working	21 (55.3%)	11 (55%)	10 (55.6%)
Relationship status	Single	7 (18.4%)	3 (15%)	4 (22%)
	Divorced/separated/widowed	9 (23.7%)	4 (20%)	5 (28%)
	Married/cohabitating	22 (57.9%)	13 (65%)	9 (50%)
Cancer treatment	Chemotherapy only	8 (21.1%)	5 (25%)	3 (16.7%)
	Hormone therapy only	16 (42.1%)	8 (40%)	8 (44.4%)
	Hormone and target therapy	8 (21.1%)	4 (20%)	4 (22.2%)
	Radiation and hormone therapy	1 (2.6%)	1 (5%)	0 (0%)
	No current medical cancer treatment	5 (13.2%)	2 (10%)	3 (16.7%)
Time since diagnosis	Years; mean (SD)	7.16 (7.18)	7.15 (7.74)	7.17 (6.72)

### Effect of VR on outcome measures

**Fig. 3:**
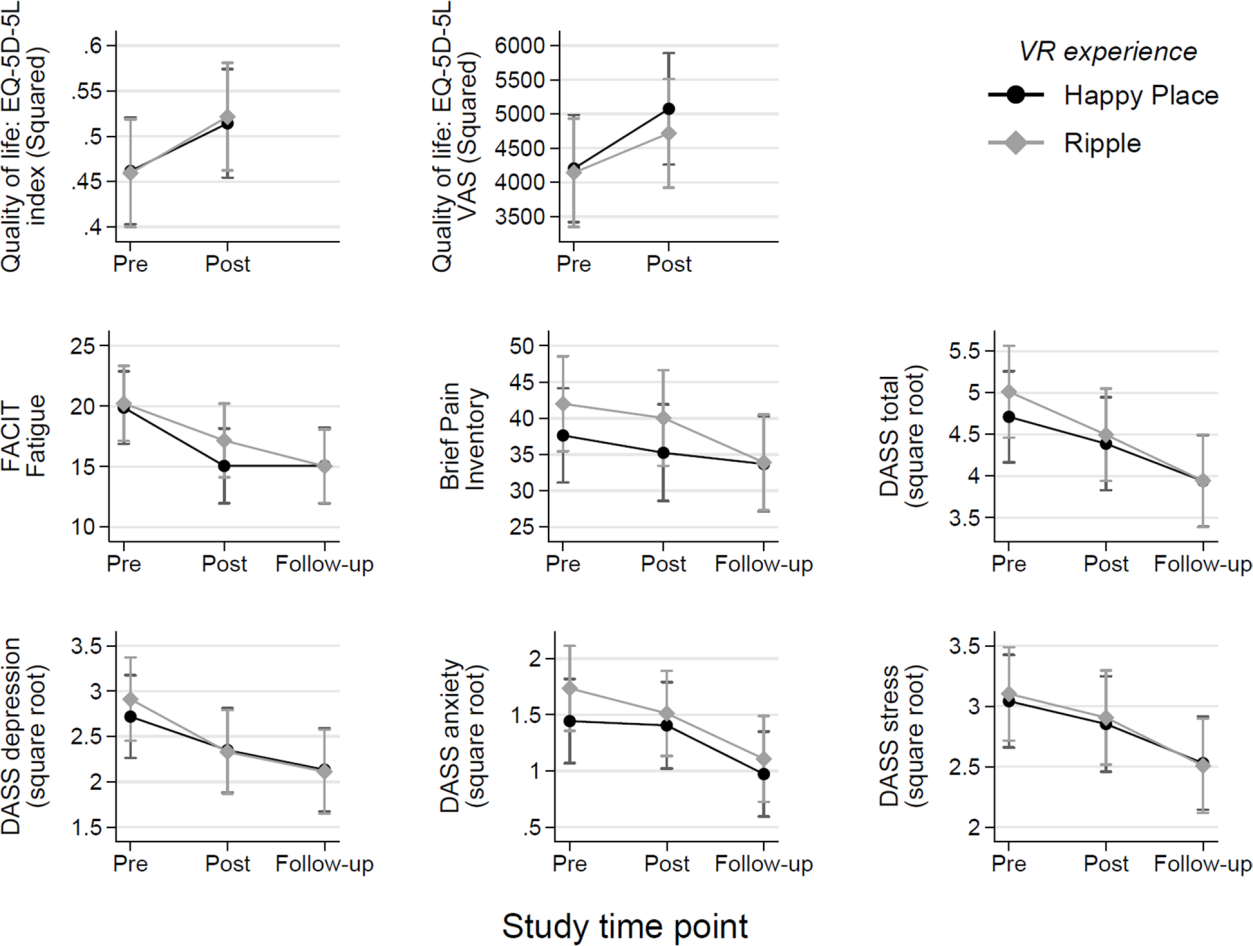
Change in Estimated Marginal Mean Outcome Measures Over the Study Period

The raw (untransformed) means for outcome measures at each time point are summarised in Table 3. A mixed model analysis showed that, overall, there was no main effect of VR experience and no interactions including VR experience; suggesting that Ripple and Happy Place experiences did not differ on any measure. This can be seen in Fig. 3, which shows the marginal means for each time point and VR experience separately, with very little difference between the two VR experiences. There were also no significant effects or interactions including study period.

**Table 3:** Descriptive Statistics of Raw Outcome Measures at Each Time Point

	Summary of outcome measure at each time point: mean (SD)					
Measure	T1 (day 0)	T2 (day 7)	T3 (day 9)	T4 (day 15)	T5 (day 21)	T6 (day 23)
EQ-Index	0.64 (0.21)	0.69 (0.17)	–	0.67 (0.21)	0.74 (0.11)	–
EQ-VAS	61.16 (21.55)	68.90 (15.24)	–	60.03 (22.96)	65.25 (23.51)	–
FACIT-Fatigue	22.41 (8.78)	17.03 (7.27)	16.52 (9.22)	18.55 (9.68)	15.48 (7.61)	14.47 (10.24)
BPI	38.61 (21.23)	39.50 (19.93)	35.51 (21.80)	39.50 (21.67)	34.25 (18.31)	30.48 (19.36)
DASS Total	33.05 (23.60)	23.42 (13.19)	20.11 (13.51)	20.69 (11.17)	20.31 (14.62)	15.94 (10.42)
DASS Depression	12.95 (11.08)	7.05 (5.73)	7.19 (6.71)	6.97 (5.12)	7.19 (6.52)	5.18 (5.02)
DASS Anxiety	5.11 (5.95)	3.63 (3.48)	2.49 (3.31)	3.14 (3.12)	3.25 (3.89)	2.12 (2.78)
DASS Stress	12.84 (8.46)	10.84 (6.34)	8.60 (5.56)	8.80 (5.14)	8.00 (6.08)	6.94 (5.29)

However, there were significant main effects of time for both VR experiences on every outcome measure, comparing baseline measurements (pre) to those taken at the end of each week-long VR experience (post) and/or 48 h later (follow-up). The estimated marginal mean pairwise comparisons for each time point are presented in detail in Table 4. There was a small but statistically significant increase in quality of life between pre- and post-intervention (note that quality of life was not measured at follow-up). Fatigue significantly decreased between the pre and post timepoints, and this was maintained at follow-up. BPI scores were lower post-intervention; however, this did not become statistically significant until follow-up. DASS Total, DASS Anxiety, and DASS Stress all decreased between pre and post, and these measures improved further and reached significance at follow-up. DASS depression also decreased between pre and post, but the further improvement at follow-up was not statistically significant.

**Table 4:** Comparison of outcome measurements across all available time points, from linear mixed models

Measure	Comparison	Estimated marginal mean difference	95% CI	*p*-value	Raw mean difference (SD)
EQ-5D-5L Index ^*a*^	Post vs Pre	0.06	[0.03, 0.09]	< 0.001	0.06 (0.13)
EQ-5D-5L VAS ^*a*^	Post vs Pre	724.65	[21.04, 1428.25]	0.044	6.78 (26.17)
FACIT-Fatigue	Post vs Pre	−3.94	[−5.83, −2.05]	< 0.001	−3.88 (7.49)
	Follow-up vs Pre	−5.00	[−7.03, −2.98]	< 0.001	−5.00 (9.10)
	Follow-up vs Post	−1.07	[−2.97, 2.83]	0.272	−1.15 (7.34)
BPI	Post vs Pre	− 2.17	[− 5.77, 1.43]	0.236	− 2.07 (15.03)
	Follow-up vs Pre	−6.01	[−10.13, − 1.90]	0.004	− 5.66 (19.89)
	Follow-up vs Post	−3.84	[−7.46, −0.22]	0.038	−4.30 (15.49)
DASS Total ^*b*^	Post vs Pre	−0.42	[−0.73, −0.11]	0.008	−5.14 (17.05)
	Follow-up vs Pre	−0.92	[−1.27, − 0.57]	< 0.001	−8.82 (15.41)
	Follow-up vs Post	−0.5	[− 0.81, − 0.19]	0.002	− 4.12 (10.46)
DASS Depression ^*b*^	Post vs Pre	− 0.48	[− 0.71, − 0.24]	< 0.001	−3.00 (7.58)
	Follow-up vs Pre	− 0.69	[− 0.96, − 0.43]	< 0.001	− 3.69 (7.03)
	Follow-up vs Post	− 0.22	[−.46, 0.02]	0.077	−1.01 (4.17)
DASS Anxiety ^*b*^	Post vs Pre	− 0.13	[− 0.39, 0.13]	0.320	− 0.66 (4.33)
	Follow-up vs Pre	−0.55	[− 0.82, − 0.28]	< 0.001	−1.80 (4.10)
	Follow-up vs Post	− 0.42	[− 0.68, − 0.16]	0.001	− 1.22 (3.16)
DASS Stress ^*b*^	Post vs Pre	−0.19	[− 0.43, 0.05]	0.117	−1.37 (6.92)
	Follow-up vs Pre	−0.55	[−0.82, − 0.29]	< 0.001	−3.07 (6.49)
	Follow-up vs Post	− 0.36	[− 0.60, − 0.12]	0.003	−1.77 (5.37)

^a^ scores analysed using squared transformation

^b^ scores analysed using square root transformation

Some of the changes over time met the published threshold for a clinically meaningful difference (MCID; Table 1). While quality of life improved by 0.06 points on the index, falling short of the MCID of 0.08 [23], the improvement of 6.78 points on the VAS was close to the clinically important difference of 7 [23]. Participants experienced an average decrease in fatigue of 5.00, larger than the MCID of 4 points [24]. The effect size (Cohen’s *d*) of the observed reduction in pain was 0.28. Since an effect size of *d* = 0.50 is suggested as the cut-off for clinical importance [25], this result was not clinically significant. The observed average reduction in depression levels of 3.80 was smaller than the suggested MCID of 5.01 [26] but, of note, it brought the average score of our participants down into the ‘normal’ range (< 9.03). The change in anxiety of − 1.89 was not deemed clinically important compared to the suggested MCID of 5.38 [26]. Finally, the observed reduction in stress scores of 3.13 was also smaller than the MCID of 4.90 [26], but again this brought our sample to within the normal range (< 12.27).

Analysis from linear mixed models (fixed effects: group, VR experience; random effect: participant) of each outcome measure at the two baselines (T1 and T4) indicated no evidence of carry-over effects, with no statistically significant interactions between period and group. Despite the successful washout period, it was clear that there were larger decreases in the outcome measures across the entire study period than across any 1 week of the study (Table 3). We therefore also looked at the change in outcome measures across the entire study period by using the difference between T1 and T6 scores (T1 and T5 for quality of life). The change in quality of life across the entire study period was 0.10 on the index, now greater than the suggested cut-off of 0.08. The change of 4.09 on the VAS did not meet the cut-off of 7. Fatigue decreased by 7.94 points overall, again larger than the MCID of 4 points. The effect size for the decrease in pain across the entire study period was *d* = 0.40 – an improvement but still short of the suggested clinically important effect size of *d* = 0.50. The gross reductions in depression (7.77) and stress (5.90) easily reached the MCID level, but the reduction in anxiety (2.99), again, did not.

### Engagement

Log books were used to record daily VR usage during the intervention weeks, with data available for 34 (89.5%) participants during the first intervention week and 31 (81.5%) during the second intervention week. Log books could not be retrieved from some participants (*n* = 4 week 1, *n* = 7 week 2), who were all in group 2 (HP:R). Where this information was available, VR usage was consistently high, with an average of 6.6 days (*SD* = 1.1, range 1–7) of VR usage recorded during each 7-day intervention week. Despite being instructed to use the VR experiences for approximately 10 min per day, the average daily duration was 12.8 min (*SD* = 5.0 min, range 6.57–35.14) and the mean number of VR usage minutes per week was 95.7 min (*SD* = 33.3 min, range 30–246). There was no difference in the duration of VR usage between the two groups in either the first (mean difference: -2.33, 95% CI: − 5.33 to 0.67, *t*(32) = 1.58, *p* = 0.124) or second (mean difference: 0.98, 95% CI: − 3.47 to 5.42, *p* = 0.656) study period.

### Acceptability

The acceptability of the interventions was high. Participants reported high likelihood of using the VR interventions again and mean ratings were similar across experiences (Mean scores: Happy Place = 73.01; Ripple = 66.67). Participant comments indicated that participants perceived the experiences to have provided benefit:

*"Since starting the experiment I have had more energy, lasted full days at work, could still function when I got home, my memory is better ... it's the best I've felt since before starting treatment."*

*"With my lack of mobility that’s resulted from my illness, I really enjoyed the VR as it made me feel like I'm not house bound... I could immerse myself elsewhere and it helped take the focus off my pain."*

However, some participants reported adverse effects in using the VR headsets including feeling *“claustrophobic”* and *“the first time I used it, I looked at a lot of things (the interactive things) and I got a bit dizzy/nauseous”.*

Feedback about specific aspects participants ‘liked’ and ‘did not like’ regarding the VR experiences fell within two key themes: 1) relaxation was helpful, and 2) it was enjoyable to spend time in nature. Theme one reflects the benefits gained from engaging in a relaxation experience. Happy Place was described as “*very similar to meditation methods*” and Ripple was “*very relaxing … , I found it much easier to get to sleep after using it*” and “*it made me stop in the day and let my mind be a lot more calm and still than it usually is”.* Key suggestions for improvement centred on inducing relaxation more effectively by keeping the experiences simple and slow paced. Examples of participant’s comments in this regard are “[there were] *too many things to interact with* [and it was] *sometimes hard to relax”, “it changed from day to night (and vice versa) too quickly”*, and “*the image cycle was too short.”*

The second theme reflects how participants enjoyed connecting with the nature settings because a) it reminded them of their childhood “*loved camping … brought back things I did as a kid … sitting by fire … it was truly beautiful*” (Happy Place), and b) it connected them with the tangible aspects of nature, “*feels like I’m inhaling pure freshness and the sound and atmosphere makes it feel so real*” and *“especially the beach … I looked forward to the time when I could chill out, felt like I was there and I loved it”* (Ripple). Connection with nature was reinforced by feedback suggesting a preference for the real nature footage of Ripple compared to the *“cartoonish”* and unrealistic graphics of Happy Place. Another person noted that “*some of the scenes made me feel a little isolated*” (Ripple) which suggests there is a balance between quiet space and feeling alone and that some interactivity can counter feelings of isolation.

## Discussion

This pilot study tested the efficacy and acceptability of an immersive VR experience used at-home by patients with MBC. Although there were no differences between interventions, scores on quality of life, fatigue, pain, depression, anxiety, and stress all improved over time between baseline and post-intervention and/or follow-up timepoints. Scores for fatigue, arguably the most important symptom [18], easily met the level considered clinically important at intervention follow-up and across the entire study period. Improvements also met the level considered clinically important across the entire study period for quality of life, depression, and stress. Depression and stress levels dropped below the clinical cut-offs after the intervention. Taken together, the results from this pilot study suggest that immersive VR can be an effective therapy for the physical and psychological symptoms of MBC and warrant further exploration in a full randomized controlled trial. This study aimed to address four key gaps in the VR research to date and these are discussed below.

First, our study demonstrated quality of life improvements via VR usage. Fifty-nine percent of surveyed women with MBC in New Zealand state that the symptoms and side-effects of their treatment negatively impact their quality of life, and only one-third feel that they have a good level of control over their symptoms [36]. We have shown that a self-reported increase in quality of life occurred alongside a decrease in the severity of symptoms such as fatigue, pain, depression, anxiety, and stress. Interestingly, only 6% of oncology therapies recently approved by the FDA were able to report a clinically important increase in quality of life [37]. Other research suggests there may be important flow on effects from increased quality of life including improved immune functioning from reduced stress and depression [9] and increased adherence to treatment due to better symptom management [11, 38]. Lower anxiety and fatigue also predict longer recurrence-free times and longer overall survival in early breast cancer [39]. Thus, our preliminary findings suggest that using VR to reduce symptoms and side-effects in patients with MBC has the potential to improve quality of life.

Second, we have demonstrated that the established benefits of VR on symptoms of pain, depression, and anxiety, also extend to fatigue. In fact, the decrease in fatigue was one of the more robust findings; this was clinically important across the average of the one-week interventions as well as across the entire three-week study period. Fatigue is rated the most important symptom by advanced cancer patients [18]. The prevalence of “severe” fatigue in patients with advanced cancer is likely to range from 60 to 90% [17, 36] and often co-occurs with pain [17]. Fatigue in cancer survivors is also associated with higher levels of pro-inflammatory cytokines [40] and chronic inflammation [41], creating an environment which promotes tumour growth [42]. Including fatigue as a measure gives us a greater understanding of how VR can improve the lives of patients with MBC in a way that is truly meaningful to them.

Third, our findings demonstrate that an at-home, patient-operated, VR intervention can be a viable and acceptable treatment for MBC patients. While the effects of VR during treatments or hospitalization had been studied, there had been no previous work investigating whether patients with MBC could use VR to deal with the day-to-day occurrence of side effects in their own homes. Accessible treatments are vital in a healthcare system where access to treatment varies dramatically between regions and regional patients must travel long distances for treatment [36]. People who live in a town or city without a tertiary cancer treatment centre typically have fewer treatment and support options and are less likely to participate in clinical trials [36]. Additionally, 15.8% of our sample were Māori women, who face disproportionately high rates of MBC diagnoses and low survival rates through restricted access to treatment [36]. Since the study was completed, the Covid-19 pandemic has further restricted healthcare and travel options for these patients, making home-based interventions even more essential. The observed effects from this at-home intervention also counter the theory that VR interventions work by removing the individual from anxiety provoking environments [15]. Previous VR studies took place in hospitals, where the surrounding environment can exacerbate anxiety and other symptoms [15]. That these effects translated to a location where participants should presumably be more relaxed suggests that virtually removing someone from an anxiety provoking situation is not the only mechanism by which VR exerts its influence.

Finally, our findings indicate the duration of beneficial effects. While there has been some work demonstrating maintenance of effects over time, this has not been well-established. One study noted that using VR during chemotherapy could reduce instances of vomiting and fatigue for up to 3 days [19], and another pilot study reported effects from 2 to 48 h after the VR intervention [20]. Other studies have provided anecdotal evidence [11] or non-significant trends [43] suggesting an extended analgesic effect. We have demonstrated that the effect of VR across multiple measures was either maintained at 48-h follow-up (in the case of fatigue and depression) or had improved further (pain, anxiety, and stress). This has implications for another of the primary cognitive explanations of how VR exerts its effect – through distraction. Immersive VR is engaging and may divert the patient’s attentional resources from focusing on pain or rumination to more helpful endeavors [11]. However, presumably this distraction effect would not occur in the days after removal of the headsets and so cannot fully explain maintenance of VR effects. Our findings suggest that the positive effects of VR interventions can be sustained for at least 48 h post-intervention, beyond the point at which distraction effects would have worn off.

### Limitations and next steps

Although this work has several strengths and extends the literature on the use of VR interventions by people with MBC, it is not without limitations. The most important issue requiring consideration is the lack of a control group which limits our ability to claim causation of effects. However, this pilot study has demonstrated beneficial effects over time and provides evidence that a follow-up randomized controlled trial is feasible, valuable, and necessary. The feedback to the open-ended questions demonstrates acceptability and informs development of future VR experiences for further research. We are also unable to quantify how much, if any, additional benefit VR may provide MBC patients over typical pain or anti-depressant medication, or alternative psychological interventions such as hypnosis, image therapy, or standard meditation and mindfulness practice. Further studies should investigate these possibilities.

Another suggestion for further studies is considering lengthening the duration of exposure to VR experiences. While most of our measures showed clinically important differences across time, this was not always the case. This may suggest that a single week of VR was not sufficient to produce clinically important improvements in some metrics, despite clearly producing statistically significant improvements. This finding fits with previous work suggesting the effects of VR do not diminish with repeated use [15] and indicates that there may be a dose-response relationship where participants gain greater benefit with increased exposure. Further studies should also consider that some people may find wearing VR headsets uncomfortable or disorienting and factor this possibility in to the development of interventions.

Furthermore, the observed reductions in anxiety did not meet the threshold for a clinically important decrease. One reason for this may be that our sample reported relatively low anxiety from the outset; the mean DASS Anxiety score of 5.11 at baseline was already well within the “normal” range (< 6.27). This may have created a floor effect in which anxiety could not have been further reduced by the intervention. The DASS anxiety subscale also displayed poor internal reliability in our sample, even after the removal of one item related to cancer treatment. Recruiting participants with high baseline anxiety for future studies would enable investigation in a group that, arguably, are in need of such an intervention.

Finally, to minimize participant burden, we did not include the quality of life measure at the 48-h follow-up. While we know that scores on all other measures improved at this time point, we are unable to comment on whether quality of life improvements were also sustained. Future studies should measure quality of life over time to see whether clinically important improvements are maintained. Another important extension would be to look for downstream effects on treatment adherence that come from being better able to tolerate the side-effects of treatment. Similarly, measuring physiological or biological outcomes could reveal a link between the effects of VR on fatigue, pain, and mood, and potentially health outcomes and survival rates—for example, an associated decrease in cortisol levels or inflammation would suggest that such VR interventions might even improve immune function and responses to treatment [42, 44] and would provide objective outcome measures rather than the self-report measures we have used.

## Conclusions

This pilot study has shown that immersive VR interventions have the potential to produce meaningful and sustained reductions in symptoms of fatigue and improve quality of life in women with MBC. Importantly, positive results were sustained for at least 2 days after people stopped using the VR headsets. Furthermore, this work indicates using a VR intervention in a patient’s own home is acceptable to patients. Our results suggest opportunities for future studies with longer duration and a wider range of outcomes including objective biometric measures.

## Data Availability

The dataset used and analysed during the current study are available from the corresponding author on reasonable request.

## Abbreviations

BPI: Brief Pain Inventory – Short Form
BCFNZ: Breast Cancer Foundation NZ
DASS: Depression, Anxiety, and Stress Scales
FACIT: Functional Assessment of Chronic Illness Therapy
FDA: Food and Drug Administration
LCD: Liquid Crystal Display
MBC: Metastatic breast cancer
MCID: Minimum clinically important difference
REML: Restricted maximum likelihood
VR: Virtual reality
